# The Role of ARNI in Enhancing Outcomes of Cardiac Resynchronization Therapy: A Comprehensive Review

**DOI:** 10.3390/jcm14082743

**Published:** 2025-04-16

**Authors:** Oana Pătru, Silvia Luca, Dragoș Cozma, Cristina Văcărescu, Simina Crișan, Mihaela Daniela Valcovici, Mirela Vîrtosu, Adrian Sebastian Zus, Constantin Tudor Luca, Simona Ruxanda Drăgan

**Affiliations:** 1 Cardiology Department, “Victor Babes” University of Medicine and Pharmacy, 2 Eftimie Murgu Sq., 300041 Timisoara, Romania; oana.patru@umft.ro (O.P.); silvia.luca0@student.umft.ro (S.L.); dragos.cozma@umft.ro (D.C.); cristina.vacarescu@umft.ro (C.V.); mihaela.valcovici@umft.ro (M.D.V.); adrian.zus@umft.ro (A.S.Z.); constantin.luca@umft.ro (C.T.L.); simona.dragan@umft.ro (S.R.D.); 2 Research Center of the Institute of Cardiovascular Diseases Timisoara, 13A Gheorghe Adam Street, 300310 Timisoara, Romania; 3 Doctoral School, “Victor Babes” University of Medicine and Pharmacy, 300041 Timisoara, Romania; daniela.cozma@umft.ro; 4 Institute of Cardiovascular Diseases Timisoara, 13A Gheorghe Adam Street, 300310 Timisoara, Romania

**Keywords:** cardiac resynchronization therapy, guideline-directed medical therapy, sacubitril/valsartan, reduced ejection fraction heart failure, left ventricle ejection fraction

## Abstract

**Background/Objectives:** Cardiac resynchronization therapy (CRT) and angiotensin receptor–neprilysin inhibitors (ARNIs) are cornerstone therapies for patients with heart failure with reduced ejection fraction (HFrEF). However, nearly 30% of patients show no significant response to CRT alone. The potential of ARNI to enhance CRT outcomes—especially in non-responders—is an emerging field of interest. The objective of this review is to systematically evaluate and synthesize the available evidence on the clinical outcomes of combining CRT with ARNI therapy in patients with HFrEF. **Methods:** We conducted a comprehensive search of PubMed, Scopus, and Google Scholar up to September 2024, using the keywords “CRT and ARNI” and “cardiac resynchronization therapy and sacubitril/valsartan”. We included retrospective and prospective clinical studies, observational studies, and review articles reporting on patients with HFrEF treated with both CRT and ARNI. Studies not in English, animal studies, and those without full-text availability were excluded. Study selection and data extraction were performed in duplicate by independent reviewers, using PRISMA guidelines for transparency. The final selection included 8 studies published in the last four years, summarized by design, population, outcomes, and statistical significance. **Results:** The reviewed studies suggest that ARNI therapy, when combined with CRT, may contribute to improvements in left ventricle ejection fraction (LVEF), NYHA functional class, and ventricular remodeling, particularly in CRT non-responders. Some studies also report a potential reduction in ventricular arrhythmias and implantable cardioverter-defibrillator (ICD) interventions. However, outcomes varied across subgroups, and the influence of ARNI timing relative to CRT implantation remains inconclusive. Limitations: Heterogeneity in study designs and small sample sizes in some included studies limited the ability to conduct a meta-analysis. This review is not registered. **Conclusions:** ARNI therapy shows promise in enhancing CRT response in patients with HFrEF, particularly in non-responders. Further large-scale, prospective studies are needed to clarify optimal patient selection and treatment sequencing.

## 1. Introduction

HF is the result of some cardiac conditions, which may cause gradual weakening or stiffening of the heart, leading to a reduced capacity to fill and pump blood effectively. HF was divided into two categories: heart failure with preserved ejection fraction (HFpEF) and HFrEF and, as of the latest guidelines in the field, an intermediate category emerging, with characteristics overlapping both HFrEF and HFpEF—heart failure with mildly reduced ejection fraction. This classification is based on the measurement of LVEF. Loss of cardiomyocytes leads to HFrEF, a condition more frequently observed in men [1,2,3]. Patients with cardiomyopathy and valvular heart disease are often hospitalized for HF, the leading cause of these admissions. This condition is linked to high mortality and morbidity rates globally [4].

Significant efforts have focused on determining the best treatment strategy for heart failure to alleviate symptoms and improve patient outcomes. Congestion-related symptoms are commonly treated with diuretics and digitalis. Diuretics help decrease the heart’s workload, while digitalis improves myocardial contractility [5]. Angiotensin receptor inhibitors, beta-blockers, ACEIs, and mineralocorticoid receptor antagonists have shown effectiveness in improving symptoms and outcomes. Studies have demonstrated these therapies enhance quality of life, reverse ventricular remodeling, and lower mortality and hospitalization rates [6].

CRT is regarded as one of the most effective cardiac remodeling therapies, surpassed only by beta-blockers [7]. It promotes reverse remodeling, decreases the size of the left ventricle and atrium, improves LVEF, and lessens the severity of functional mitral regurgitation. These benefits have been associated with favorable clinical outcomes in the MADIT-CRT and REVERSE trials [8].

Moreover, recent observations indicate that ARNI provides superior cardiovascular protection for HF patients compared to traditional therapies [9]. The combination ARNI has shown effectiveness in improving hemodynamic and neurohormonal outcomes in HF cases. A study revealed that this drug outperformed angiotensin-converting enzyme inhibitor (ACEi) in reducing the risk of cardiovascular death or hospitalization among HFrEF patients [10].

The PROVE-HF trial [11] recently highlighted ARNI’s effects on cardiac remodelling, revealing a 9.4% increase in LVEF over a 12-month period, along with reductions in the left ventricular end-diastolic and end-systolic volume indices, as well as the left atrial volume index. Additionally, the Italian observational study SAVE-ICD, led by Federico Guerra, found that following six months of ARNI therapy, 25% of patients implanted with ICDs for primary prevention reached an LVEF of 35% or higher [12].

The CeRtiTude cohort study, conducted more recently, found comparable rates of sudden cardiac death (SCD) between CRT-P and CRT-D recipients over a two-year follow-up, despite the CRT-P group being older, having poorer health status, and presenting with more comorbid conditions [13]. A 2019 analysis by Barra et al. [14] indicated that the risk of SCD in patients undergoing CRT has dropped by over four times in the last two decades, with a more pronounced reduction observed among those treated with CRT-P. This reduction was linked to increased LVEF, greater beta-blocker use, reduced QRS duration, and less frequent use of antiarrhythmic drugs [14]. The PARADIGM trial showed a 22% reduction in SCD rates [2]. Studies by de Diego et al. [15] and Martens et al. [16] found that ARNI significantly reduced sustained and non-sustained ventricular arrhythmias, appropriate ICD shocks, and improved pacing parameters in ICD patients on ARNI [17]. ARNI reduces arrhythmia burden mainly by enhancing cardiac remodeling, though smaller studies have also shown reductions in QRS duration, QTc interval, and mechanical dispersion, evaluated through LV global longitudinal strain imaging [18]. ARNI’s beneficial influence on electrophysiological parameters is thought to be mediated primarily through reverse cardiac remodeling, potentially reducing fibrosis and scar burden—key contributors to electrical dys-synchrony and CRT non-response. Fibrosis and increased myocardial scar tissue disrupt the integrity of the conduction system, resulting in heterogeneous electrical conduction and impaired synchronization. In this context, ARNI therapy has demonstrated efficacy in modulating myocardial fibrosis and reducing adverse electrical remodeling. For instance, ARNI treatment in CRT non-responders was associated with significant improvements in left ventricular ejection fraction (LVEF) and ventricular volumes, as well as modulation of microRNAs linked to fibrosis, apoptosis, and hypertrophy. These findings underline ARNI’s capability to enhance the CRT response by ameliorating electrical conduction abnormalities, improving myocardial electrophysiological homogeneity, and potentially reversing structural myocardial damage, thus offering a promising pharmacologic adjunct to enhance CRT outcomes.

The 2023 Focused Updates on the 2021 ESC Guidelines for the diagnosis and treatment of acute and chronic heart failure reinforce the importance of CRT and and guideline-directed medical therapy GDMT in HF management. CRT remains a Class I recommendation for patients with HFrEF (LVEF ≤ 35%), sinus rhythm, and QRS ≥ 150 ms (especially with LBBB morphology) who remain symptomatic despite optimal GDMT. Early CRT implantation is now emphasized in LBBB-induced cardiomyopathy, particularly in patients with a poor response to medical therapy. Additionally, CRT in atrial fibrillation patients with HFrEF is beneficial if a high percentage (>95%) of biventricular pacing can be achieved, with AV node ablation considered in cases of inadequate pacing. The GDMT recommendations have been strengthened, particularly with ARNI now preferred over ACEi and ARBs in HFrEF patients (Class I), while SGLT2 inhibitors (dapagliflozin and empagliflozin) maintain a strong Class I recommendation for all HFrEF patients, regardless of diabetes status. Beta-blockers and MRAs continue to be Class I recommendations, while diuretics are used only for symptom relief and congestion management. Notably, quadruple therapy (ARNI + beta-blocker + MRA + SGLT2 inhibitor) is now recommended within 4 weeks for eligible patients, reflecting a shift toward early, aggressive medical optimization. Overall, GDMT remains the foundation of HF management, with CRT integrated early in eligible patients, ensuring an optimal balance between pharmacologic and device-based therapies [19].

**Figure 1:**
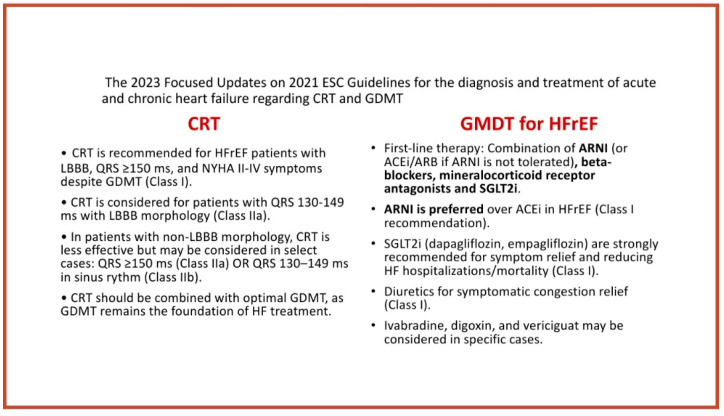
The 2023 Focused Updates on 2021 ESC Guidelines for the diagnosis and treatment of acute and chronic heart failure regarding CRT and GDMT [19].

The diagram below illustrates the ESC Guidelines for HF regarding CRT and GDMT (Figure 1) [19].

This article aims to evaluate the effectiveness of combining ARNI therapy with CRT in individuals diagnosed with HFrEF.

## 2. Materials and Methods

We systematically conducted a comprehensive literature search, targeting multiple published study designs, including retrospective studies, prospective studies, and review articles. Our search encompassed widely recognized databases, specifically PubMed, Google Scholar, and Scopus, using relevant keyword combinations: “CRT and ARNI” or “Cardiac resynchronization therapy and sacubitril/valsartan”. To ensure methodological rigor and transparency, the study selection process adhered strictly to the Preferred Reporting Items for Systematic Reviews and Meta-Analyses (PRISMA) guidelines.

In addition to electronic database searches, manual searches were meticulously conducted to enhance the identification of potentially relevant studies. PubMed searches specifically utilized Medical Subject Headings (MeSH) terms to efficiently identify articles associated with CRT, ARNI, and HFrEF therapy.

Studies were excluded if they were not available in the English language, involved animal subjects rather than humans, represented duplicate publications, or consisted solely of abstracts without accessible full-text articles. The literature search was completed in September 2024 and was intentionally unrestricted regarding the publication year to capture all relevant historical and contemporary studies. The search and subsequent screening processes were independently performed by cardiologists specialized in heart failure management to ensure the inclusion of pertinent and high-quality research.

Initially, a manual screening was performed by reviewing article titles with explicit reference to the keywords “CRT and ARNI” and “Heart failure therapy.” Following this, an advanced PubMed search was executed using the MeSH term “CRT and ARNI” [Mesh] to capture additional eligible studies comprehensively. All qualifying articles identified during this thorough selection process were systematically documented in a structured Microsoft Excel table (version 2408), featuring detailed information including the title of the study, names of authors, year of publication, journal name, and the specific type of publication. Study selection and data extraction were performed in duplicate by independent reviewers.

A simplified risk of bias analysis was performed using modified criteria based on the Newcastle–Ottawa Scale, appropriate for the predominantly observational and retrospective nature of the included studies. Each study was assessed for selection bias, outcome/exposure clarity, and follow-up adequacy. The overall risk of bias was rated as low, moderate, or high. Most included studies were of moderate quality, with some limitations related to small sample size, non-randomized design, and lack of comparability between groups. These limitations were acknowledged in the interpretation of findings. Another limitation was the heterogeneity in study designs and small sample sizes in some included studies, which limited the ability to conduct a meta-analysis. The review was initiated retrospectively, and a formal protocol had not been developed or submitted for registration prior to the literature search and data synthesis. While registration is considered best practice, the review was conducted with adherence to PRISMA 2020 guidelines to ensure methodological transparency and rigor.

**Figure 2:**
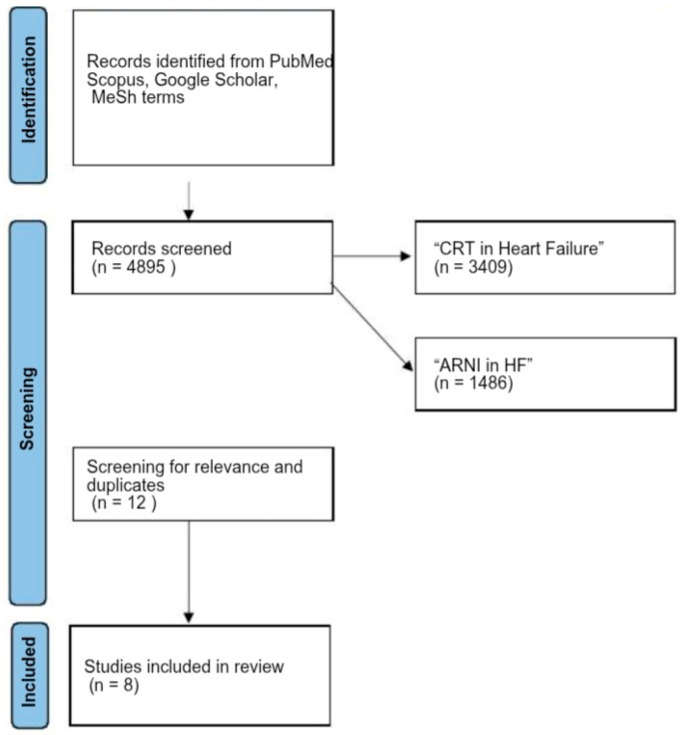
Flow diagram of the review process.

The diagram illustrates the methodology used by the authors for selecting articles, created using Microsoft Office suite. A total of 3409 articles were identified through search engines using the keywords “CRT in heart failure” and 1486 articles using “ARNI in HF” or “angiotensin receptor-neprilysin inhibitor in HF”. After searching articles that comprised both “CRT and ARNI in HF” keywords and removing duplicates and excluding articles that were irrelevant or failed to meet the predefined inclusion criteria, the final selection comprised 8 articles (Figure 2).

## 3. Results

The table below summarizes key studies published over the past four years, detailing their titles, authors, publication year, patient population, and main findings (Table 1).

### 3.1. Timing of ARNI Therapy in Relation to CRT

A total of 258 patients were enrolled in this study, of whom 202 (78%) were male, with a mean age of 69.4 years. Among these individuals, the majority (67.4%) had an ischemic etiology of HF with reduced ejection fraction. Additionally, a substantial proportion of these patients presented with multiple comorbidities, including diabetes mellitus (68%), dyslipidemia (87%), and arterial hypertension (73%), reflecting the high cardiovascular risk burden in this population. Regarding the response to CRT, fifty-two percent (133 patients) were classified as CRT responders, demonstrating an improvement in left ventricular function following device implantation. However, when comparing patients who received ARNI and/or SGLT2i with those who did not, there was no statistically significant difference in the proportion of CRT responders between the two groups (55% vs. 47%; *p* = not significant). Similarly, the mean increase in LVEF was comparable between patients who received ARNI or SGLT2i therapy and those who did not, with no significant variation observed (+7.7% vs. +10.3%; *p* = not significant). Furthermore, within the subgroup of patients who were prescribed ARNI or SGLT2i, no significant differences were identified between those who had initiated these therapies prior to CRT implantation and those who commenced treatment afterward. This suggests that, in this cohort, the timing of ARNI or SGLT2i introduction did not appear to significantly influence the likelihood of CRT response or improvements in LVEF. These findings underscore the need for further research to elucidate the potential interaction between pharmacologic therapy and device-based interventions in patients with HFrEF. [22].

Another study specifically examined the potential benefits of CRT in patients diagnosed with LBBB-induced cardiomyopathy, a condition characterized by electromechanical dys-synchrony that contributes to progressive left ventricular dysfunction and adverse remodeling. The findings suggest that early CRT implantation in this subset of patients is associated with significant improvements in LVEF and functional capacity, with a proportion of individuals experiencing complete normalization of LVEF, effectively reversing their cardiomyopathy. This hyper-response phenomenon highlights the potential of CRT to correct conduction abnormalities, restore synchronous ventricular contraction, and promote favorable cardiac remodeling, particularly in patients without extensive structural myocardial damage. Conversely, in patients with LBBB-induced cardiomyopathy, optimal medical therapy (OMT) based on current clinical guidelines does not appear to consistently induce significant improvements in LVEF or functional class, raising the question of whether pharmacologic treatment alone is sufficient for managing this condition. While GDMT remains the cornerstone of HF treatment, its efficacy in modifying disease progression in LBBB-related cardiomyopathy seems to be limited, reinforcing the role of early CRT intervention in appropriately selected cases. The timing of CRT implantation in relation to OMT continues to be a topic of active investigation, with ongoing research seeking to determine the optimal therapeutic sequence that maximizes patient outcomes. Whether early CRT implantation should be prioritized over prolonged attempts at pharmacologic optimization remains an open question, as studies continue to assess the relative impact of timely device-based intervention versus extended medical management. Further clinical trials are needed to clarify which patient subgroups may benefit most from early CRT implantation, particularly in cases where OMT alone fails to yield meaningful improvements in LV function and HF symptoms. [25].

### 3.2. Impact of ARNI and SGLT2i on CRT Outcomes

A retrospective study investigating the outcomes of 183 patients with HFrEF undergoing CRT sought to determine the impact ARNI SGLT2i on cardiovascular and all-cause mortality. The study cohort included a diverse population of ischemic and non-ischemic HFrEF patients, with a median age of 73 years (interquartile range 65–80 years) and 148 males (80.9%). A majority of patients (117, 74%) were prescribed ARNI (*n* = 61, 52.1%), SGLT2i (*n* = 7, 6%), or a combination of both (*n* = 49, 41.9%) and were classified under the ARNI/SGLT2i group. During a median follow-up period of 5.67 years (95% CI, 5–6.5 years), a total of 63 patients (34.4%) died, with the primary cause of death being cardiovascular-related HF in 74.6% of cases. Specifically, 44 deaths occurred in the no-drugs group, with 35 cases (79%) attributed to HF-related mortality, whereas only 19 deaths occurred in patients treated with ARNI and/or SGLT2i, among whom 12 cases (63%) were HF-related. In univariable analysis, treatment with ARNI and/or SGLT2i was associated with a reduction in all-cause mortality (hazard ratio [HR] 0.59, *p* = 0.056) and cardiovascular mortality (HR 0.47, *p* = 0.029), suggesting a potential protective effect of these medications on long-term survival in patients with CRT and HFrEF. However, following multivariable adjustment for key clinical and demographic covariates—including age, one-year CRT response, baseline LVEF, atrial fibrillation status, glomerular filtration rate, and chronic obstructive pulmonary disease—the mortality reduction benefits previously attributed to ARNI and SGLT2i diminished. Among all analyzed factors, the most significant prognostic indicator for reduced mortality risk was a positive response to CRT at one year, reaffirming the pivotal role of CRT efficacy in determining long-term survival outcomes. These findings suggest that while ARNI and SGLT2i therapy may contribute to improved HF management, their direct impact on enhancing CRT response and mortality reduction remains influenced by patient-specific variables. Moreover, the timing of ARNI/SGLT2i initiation relative to CRT implantation did not appear to significantly alter CRT response rates or survival outcomes, emphasizing the need for further studies to elucidate the synergistic potential of pharmacological and device-based therapies in HFrEF patients undergoing CRT [26].

### 3.3. ARNI in CRT Non-Responders

Given that nearly 30% of patients do not respond favorably to CRT, ARNI has been explored as a potential modifier of outcomes. In a cohort of CRT non-responders, ARNI therapy initiated six months post-implantation resulted in significant improvements over six months, similarly to what was observed in the general HF patient cohort with no CRT. Both groups showed significant improvements in LVEF and NYHA class. In CRT non-responders, LVEF increased from 25.25% to 29.5% (*p* < 0.001), NYHA class improved from 2.74 to 2.03 (*p* < 0.001), and NT-proBNP levels significantly decreased from 3884 to 2676 pg/mL, (*p* < 0.001). These effects were comparable to those observed in general HF patients without CRT, suggesting that ARNI therapy independently contributes to cardiac function recovery in CRT non-responders [20].

Data from a prospective, multicenter study evaluating ARNI in CRTd non-responders (patients receiving CRT with a defibrillator but failing to achieve expected benefits) also found improvements in cardiac remodeling markers, with a significant proportion of patients (34.9%) converting to CRT responders compared to only 6.4% in the non-ARNI group. This study revealed significant improvements in LVEF, LVESV, and functional capacity. Among ARNI users, LVEF increased significantly (*p* < 0.01) along with improved six-minute walking test (6MWT) performance. Additionally, ARNI therapy modulated key microRNAs (miR-18, miR-145, and miR-181) associated with cardiac fibrosis, apoptosis, and hypertrophy. At one-year follow-up, 34.9% of ARNI users transitioned to CRT responders, compared to only 6.4% of non-ARNI users, indicating that ARNI enhances anti-remodeling effects and functional recovery in CRT patients [23].

### 3.4. Potential Reduction in the Need for Device Therapy with ARNI

Recent studies indicate a growing body of evidence supporting the idea that integrating ARNI into OMT could potentially reduce the reliance on device-based therapy, particularly in patients with milder forms of HFrEF. This hypothesis is grounded in the well-documented ability of ARNI to promote reverse cardiac remodeling, enhance LVEF, and significantly lower hospitalization rates due to HF exacerbations. These effects suggest that, in some cases, early and aggressive medical management with ARNI may be sufficient to stabilize or even improve cardiac function, thereby delaying the need for CRT or, in select cases, rendering its implantation unnecessary. Additionally, findings from clinical trials and real-world data underscore the potential of ARNI therapy to modify disease progression in HFrEF, particularly in patients with LBBB-induced cardiomyopathy, where the interplay between conduction abnormalities and LV dysfunction is a key determinant of CRT candidacy. By improving myocardial efficiency and reducing ventricular dilation, ARNI therapy may allow a subset of patients to achieve significant functional recovery without requiring biventricular pacing. However, while these observations are promising, further longitudinal studies and randomized clinical trials are needed to determine the precise role of ARNI in influencing CRT decision-making, particularly in defining which patient populations may benefit most from a pharmacologic-first approach before considering device implantation [27].

## 4. Discussion

The findings from these studies suggest that while ARNI and SGLT2i may provide significant therapeutic benefits when used in combination with CRT, their impact on long-term survival and cardiac function improvement appears to be modulated by multiple patient-specific factors. One of the most critical determinants influencing these outcomes is the individual response to CRT, which has been identified as a key prognostic factor in HF management. This underscores the complex and multidimensional nature of HF treatment, particularly in patients with HFrEF undergoing device-based therapy, where a personalized approach to treatment is necessary to optimize clinical outcomes.

A significant proportion of HFrEF patients do not respond optimally to CRT alone, highlighting the need for complementary pharmacological strategies to enhance reverse remodeling and functional recovery. Among these, ARNI therapy has demonstrated promise in improving LVEF and functional status in CRT non-responders, suggesting that this approach may provide additional benefits beyond conventional GDMT. However, an important consideration emerging from the literature is the lack of significant differences in cardiac outcomes between patients who received ARNI and/or SGLT2i prior to CRT implantation versus those who initiated therapy after device implantation. This observation challenges previous assumptions regarding the optimal timing of pharmacologic therapy in relation to CRT and suggests that the beneficial effects of ARNI and SGLT2i may not be time-dependent but rather contingent on the patient’s underlying cardiac condition, CRT response, and overall disease progression.

The role of CRT in LBBB-induced cardiomyopathy remains well-established, with early intervention in these patients being consistently associated with improved LVEF and functional capacity. This reinforces the importance of timely diagnosis and appropriate patient selection for CRT implantation, as delayed intervention may limit the extent of cardiac reverse remodeling and symptom improvement. Furthermore, recent discussions in the field have raised the possibility that ARNI therapy could reduce the need for CRT and other device-based therapies in select HFrEF patients, particularly in those with less severe conduction abnormalities or those who demonstrate significant improvements in cardiac function with medical therapy alone. This therapeutic paradigm shift suggests a potential role for early pharmacologic optimization before proceeding with CRT implantation, an area that warrants further large-scale clinical trials to establish more definitive treatment algorithms.

Despite the increasing volume of studies supporting the use of ARNI therapy in CRT non-responders, the level of efficacy appears to vary based on patient characteristics, baseline LV function, and disease progression at the time of intervention. Additionally, the studies reviewed highlight the need for longer follow-up durations to assess whether ARNI therapy can consistently enhance CRT outcomes or potentially serve as an alternative to device therapy in specific subsets of patients. While CRT remains a cornerstone of treatment in appropriately selected patients, the integration of advanced pharmacological agents such as ARNI and SGLT2i into HF management continues to evolve. Future randomized controlled trials with extended follow-up periods will be crucial in determining whether a combination of medical and device-based therapy provides superior outcomes or if a medical-first approach may be viable in specific clinical scenarios.

Historically, patients with HF receiving CRT were primarily managed with ACEi or ARBs. Landmark CRT trials in the pre-ARNI era, such as MADIT-CRT [28], COMPANION [29], and CARE-HF [30], demonstrated significant benefits including improved quality of life, decreased mortality, and reduced hospitalization rates for patients receiving CRT along with a conventional neurohormonal blockade (ACEi/ARBs, MRAs, and beta-blockers). Studies conducted in the ARNI era, including subgroup analyses of the PARADIGM-HF [2] and PROVE-HF [31] trials, showed that ARNI administration provided superior clinical outcomes compared to ACEi or ARBs alone. When comparing CRT-supported HF patients receiving ARNI versus those treated with ACEi or ARBs, studies suggest synergistic benefits. CRT combined with ARNI therapy has shown superior improvements in ventricular function and patient outcomes compared to CRT combined with ACEi or ARB therapy alone. This combined therapy enhances the benefits originally documented with CRT, including more pronounced reverse remodeling and better clinical prognosis. Thus, contemporary evidence increasingly supports the combined use of CRT and ARNI therapy as an optimal strategy for selected patients with HF, significantly surpassing outcomes observed in earlier studies using CRT plus ACEi or ARBs alone.

An area warranting further investigation is the impact of peri-procedural complications, such as contrast-induced nephropathy (CIN) on CRT outcomes. Strisciuglio et al. [32] reported that CIN following CRT implantation can impair the recovery of LVEF in responders. While our review centers on ARNI’s role in enhancing CRT response, acknowledging and mitigating such complications is crucial for optimizing patient outcomes. Future research should aim to elucidate strategies to prevent CIN and assess its long-term impact on CRT efficacy.

These findings emphasize the critical importance of individualized treatment strategies, where the timing, sequencing, and combination of CRT and medical therapy should be tailored to maximize patient outcomes while minimizing unnecessary interventions. Further research is needed to refine patient selection criteria, optimize treatment algorithms, and determine the best approaches to integrating pharmacologic advancements with device-based therapies in HFrEF management.

Ultimately, a subject deserving further investigation is the potential of ARNI to influence electrophysiological mechanisms underlying CRT response. While preliminary findings suggest beneficial effects on electrical remodeling and conduction system parameters, more robust, large-scale studies are needed to fully elucidate ARNI’s role in modulating these processes. This represents a promising direction for future research, which may help refine patient selection and improve CRT response rates.

## 5. Conclusions

CRT and ARNI therapy are both recognized as cornerstone treatments for patients with HFrEF, each addressing distinct pathophysiological mechanisms and requiring careful patient selection to maximize therapeutic benefits. While CRT primarily functions by mechanically synchronizing ventricular contractions, thereby improving left ventricular efficiency and overall cardiac performance, ARNI therapy exerts its effects through neurohormonal modulation, enhancing natriuretic peptide activity while simultaneously inhibiting the renin–angiotensin–aldosterone system (RAAS). The complementary nature of these two interventions underscores the importance of a tailored, patient-specific approach in HF management, ensuring that individuals receive the most appropriate combination of device-based and pharmacologic therapy.

Given the heterogeneity of HFrEF populations, identifying specific subgroups of patients who derive the greatest benefit from CRT-ARNI combination therapy remains a key clinical objective. Future research efforts will be critical in refining patient selection criteria and optimizing treatment algorithms. The ultimate goal is to enhance long-term survival, reduce heart failure-related hospitalizations, and improve overall quality of life in affected individuals. Ongoing large-scale randomized trials, with extended follow-up periods, such as LIFE [33] and PARAGLIDE-HF [34], will be instrumental in providing high-quality evidence to confirm and further clarify the role of CRT and ARNI integration in HF management, helping to establish standardized guidelines for determining the optimal timing, sequencing, and patient selection for this combined therapeutic approach.

Another crucial aspect of optimizing HF therapy is determining the most effective integration of pharmacologic and device-based interventions. While CRT has been proven to significantly improve outcomes in patients with electrical dys-synchrony, the addition of ARNI and SGLT2i therapies has demonstrated a reduction in mortality and an improvement in cardiac function, particularly in patients who already exhibit a favorable response to CRT. However, findings also suggest that the precise timing of ARNI or SGLT2i initiation may not be as critical as ensuring continuous adherence to optimal medical therapy, emphasizing the importance of long-term treatment persistence and follow-up rather than immediate initiation post-CRT implantation.

Despite the increasing number of studies examining CRT and ARNI therapy independently, there has been no prior review that comprehensively synthesizes the evidence on their combined use in HF treatment within a single discussion. The integration of pharmacologic and device-based therapies remains an evolving field, and future advancements will likely focus on developing hybrid treatment approaches that maximize synergistic benefits while tailoring interventions to individual patient needs. Further long-term studies, registries, and real-world data analyses will be essential to establishing best practices, refining treatment guidelines, and ensuring that each patient receives the most effective therapeutic combination to manage HFrEF effectively.

## Figures and Tables

**Table 1:** Articles published in the last 4 years about CRT and ARNI. Abbreviations: Pts no, patients number; CRT, cardiac resynchronization therapy; LVEF, left ventricular ejection fraction; ARNI, angiotensin receptor/neprilysin inhibitor; HFrEF, heart failure with reduced ejection fraction; NTpro-BNP, N-terminal prohormone of brain natriuretic peptide; OMT, optimal medical therapy; miRs, microRNAs.

Title	Authors	Year	Pts No	Study Type	Conclusion	Key Clinical Endpoints Statistical Significance
The effectiveness of ARNI medication in patients, non-responder to cardiac resynchronization therapy	Szabo et al. [20]	2022	205	Observational	ARNI improved LVEF, HF biomarker level, and functional class in CRT-NR patients.	LVEF ↑ from 25.25% to 29.5% (*p* < 0.001), NYHA class improved (*p* < 0.001) ProBNP ↓ (*p* < 0.001)
Impact of sacubitril/valsartan and gliflozins on cardiac resynchronization therapy response in ischemic and non-ischemic heart failure patients	Fonderico et al. [21]	2023	178	Case Control	ARNI alone or with SGLT2i improved CRT response in ischemic patients more than non-ischemic patients.	CRT response ↑, LVEF ↑ (no specific *p*-value reported)
Response to cardiac resynchronization therapy and treatment with ARNi and iSGLT2	Fonderico et al. [22]	2023	259	Retrospective	Timing of ARNI/iSGLT2 did not affect CRT response.	LVEF ↑ 7.7% vs. 10.3% (NS), CRT response 55% vs. 47% (NS)
Angiotensin receptor/Neprilysin inhibitor effects in CRTd non-responders: From epigenetic to clinical beside	Sardu et al. [23]	2022	418	Observational	ARNI modulated miRs and improved remodeling in CRT-D NR.	LVEF ↑ (*p* < 0.01), 6MWT ↑, 34.9% converted to responders vs. 6.4% (*p* < 0.01), ↓ miR-181 level and inflammatory markers ↑ miR-18 snd miR-145 level
Defining the gap in heart failure treatment in patients with cardiac implantable electronic devices	Salimian et al. [24]	2023	27	Retrospective	Significant GDMT gaps remain in HF patients with CRT.	Prescription and titration rates reported (no statistical endpoint included)
Should cardiac resynchronization therapy be prescribed before optimizing medical therapy in patients with left bundle branch block-induced cardiomyopathy?	Jorge Toquero Ramos [25]	2023	21	Retrospective Observational	CRT led to LVEF normalization in LBBB cardiomyopathy; OMT did not.	LVEF ↑ (exact *p*-value not reported), functional class ↑
A time-dependent analysis of sacubitril/valsartan and gliflozins benefits in patients receiving cardiac resynchronization therapy	Faccenda et al. [26]	2024	183	Retrospective	ARNI/SGLT2i associated with lower all-cause and CV mortality but has not shown any further improvement in survival outcomes when compared with CRT alone.	CV mortality ↓ (HR 0.47, *p* = 0.029), all-cause mortality ↓ (HR 0.59, *p* = 0.056), CRT response major predictor
Will introduction of ARNI reduce the need of device therapy in heart failure with reduced ejection fraction?	Kaur et al. [27]	2021	-	Review	ARNI and CRT are complementary; ARNI may reduce CRT needs in some cases.	Narrative synthesis, no clinical data presented

## Data Availability

No new data were created or analyzed in this study. Data sharing is not applicable to this article.

## References

[1] Levy W.C. (2017). Should nonischemic CRT candidates receive CRT-P or CRT-D?. J. Am. Coll. Cardiol..

[2] Mcmurray J.J.V., Packer M., Desai A.S., Gong J., Lefkowitz M.P., Rizkala A.R., Rouleau J.L., Shi V.C., Solomon S.D., Swedberg K. (2014). Angiotensin-neprilysin inhibition versus enalapril in heart failure. N. Engl. J. Med..

[3] Murphy S.P., Ibrahim N.E., Januzzi J.L. (2020). Heart failure with reduced ejection fraction: A review. JAMA.

[4] Groenewegen A., Rutten F.H., Mosterd A., Hoes A.W. (2020). Epidemiology of heart failure. Eur. J. Heart Fail..

[5] Parmley W.W. (1992). Pathophysiology of congestive heart failure. Clin. Cardiol..

[6] Bao J., Kan R., Chen J., Xuan H., Wang C., Li D., Xu T. (2021). Combination pharmacotherapies for cardiac reverse remodeling in heart failure patients with reduced ejection fraction: A systematic review and network meta-analysis of randomized clinical trials. Pharmacol. Res..

[7] De Diego C., Gonzalez-Torres L., Nunez J.M., Carrasco R., Almendral J. (2018). Advances in pharmacological therapy in reduced left ventricular heart failure patients with implantable cardiac defibrillator and cardiac resynchronization. Clin. Cardiol. J..

[8] Linde C., Ellenbogen K., McAlister F.A. (2012). Cardiac resynchronization therapy (CRT): Clinical trials, guidelines, and target populations. Heart Rhythm.

[9] Kim H.M., Hwang I.-C., Choi W., Yoon Y.E., Cho G.-Y. (2021). Combined effects of ARNI and SGLT2 inhibitors in diabetic patients with heart failure with reduced ejection fraction. Sci. Rep..

[10] Kuchulakanti P.K. (2020). ARNI in cardiovascular disease: Current evidence and future perspectives. Future Cardiol..

[11] Januzzi J.L., Prescott M.F., Butler J., Felker G.M., Maisel A.S., McCague K., Camacho A., Piña I.L., Rocha R.A., Shah A.M. (2019). Association of change in N-terminal pro-B-type natriuretic peptide following initiation of sacubitril-valsartan treatment with cardiac structure and function in patients with heart failure with reduced ejection fraction. JAMA.

[12] Guerra F., Ammendola E., Ziacchi M., Aspromonte V., Pellegrino P.L., Del Giorno G., Dell’Era G., Pimpini L., Santoro F., Floris R. (2021). Effect of SAcubitril/Valsartan on left vEntricular ejection fraction and on the potential indication for Implantable Cardioverter Defibrillator in primary prevention: The SAVE-ICD study. Eur. J. Clin. Pharmacol..

[13] Marijon E., Leclercq C., Narayanan K., Boveda S., Klug D., Lacaze-Gadonneix J., Defaye P., Jacob S., Piot O., Perier M.-C. (2015). Causes-of-death analysis of patients with cardiac resynchronization therapy: An analysis of the CeRtiTuDe cohort study. Eur. Heart J..

[14] Barra S., Providência R., Narayanan K., Boveda S., Duehmke R., Garcia R., Leyva F., Roger V., Jouven X., Agarwal S. (2019). Time trends in sudden cardiac death risk in heart failure patients with cardiac resynchronization therapy: A systematic review. Eur. Heart J..

[15] de Diego C., Gonzalez-Torres L., Núñez J.M., Inda R.C., Martin-Langerwerf D.A., Sangio A.D., Chochowski P., Casasnovas P., Blazquez J.C., Almendral J. (2018). Effects of angiotensin-neprilysin inhibition compared to angiotensin inhibition on ventricular arrhythmias in reduced ejection fraction patients under continuous remote monitoring of implantable defibrillator devices. Heart Rhythm.

[16] Martens P., Nuyens D., Rivero-Ayerza M., Van Herendael H., Vercammen J., Ceyssens W., Luwel E., Dupont M., Mullens W. (2019). Sacubitril/valsartan reduces ventricular arrhythmias in parallel with left ventricular reverse remodeling in heart failure with reduced ejection fraction. Clin. Res. Cardiol..

[17] Vecchi A.L., Abete R., Marazzato J., Iacovoni A., Mortara A., De Ponti R., Senni M. (2020). Ventricular arrhythmias and ARNI: Is it time to reappraise their management in the light of new evidence?. Heart Fail. Rev..

[18] Valentim Gonçalves A., Pereira-da-Silva T., Galrinho A., Rio P., Moura Branco L., Soares R., Feliciano J., Ilhão Moreira R., Cruz Ferreira R. (2019). Antiarrhythmic effect of sacubitril-valsartan: Cause or consequence of clinical improvement?. J. Clin. Med..

[19] McDonagh T.A., Metra M., Adamo M., Gardner R.S., Baumbach A., Böhm M., Burri H., Butler J., Čelutkienė J., Chioncel O. (2023). 2023 Focused Update of the 2021 ESC Guidelines for the diagnosis and treatment of acute and chronic heart failure: Developed by the task force for the diagnosis and treatment of acute and chronic heart failure of the European Society of Cardiology (ESC) with the special contribution of the Heart Failure Association (HFA) of the ESC. Eur. Heart J..

[20] Szabo K., Sandorfi G., Nagy L.T., Clemens M., Toth A., Borbely A., Polik Z.S., Raduly A., Csanadi Z. (2022). The effectiveness of ARNI medication in patients, non-responder to cardiac resynchronization therapy. Eur. Heart J..

[21] Fonderico C., Pergola V., Faccenda D., Salucci A., Comparone G., Marrese A., Ammirati G., Cocchiara L., Varriale A., Esposito G. (2023). Impact of sacubitril/valsartan and gliflozins on cardiac resynchronization therapy response in ischemic and non-ischemic heart failure patients. Int. J. Cardiol..

[22] Fonderico C., Faccenda D., Pergola V., Marrese A., Comparone G., Meola M., Salucci A., Cocchiara L., Addeo L., Ammirati G. (2023). Response to cardiac resynchronization therapy and treatment with ARNi and iSGLT2. Europace.

[23] Sardu C., Sardu C., Massetti M., Massetti M., Scisciola L., Scisciola L., Trotta M.C., Trotta M.C., Santamaria M., Santamaria M. (2022). Angiotensin receptor/Neprilysin inhibitor effects in CRTd non-responders: From epigenetic to clinical beside. Pharmacol. Res..

[24] Salimian S., Moghaddam N., Deyell M.W., Virani S.A., Bennett M.T., Krahn A.D., Andrade J.G., Hawkins N.M. (2023). Defining the gap in heart failure treatment in patients with cardiac implantable electronic devices. Clin. Res. Cardiol..

[25] Ramos J.T. (2023). Should cardiac resynchronization therapy be prescribed before optimizing medical therapy in patients with left bundle branch block-induced cardiomyopathy?. Rev. Española Cardiol. (Engl. Ed.).

[26] Faccenda D., Pergola V., Salucci A., Marrese A., Comparone G., Cocchiara L., Fonderico C., Ammirati G., Muoio M., Grimaldi A. (2024). A time-dependent analysis of sacubitril/valsartan and gliflozins benefits in patients receiving cardiac resynchronization therapy. Europace.

[27] Kaur N., Pruthvi C., Rohit M. (2021). Will introduction of ARNI reduce the need of device therapy in heart failure with reduced ejection fraction?. Egypt. Heart J..

[28] Breithardt G. (2009). MADIT-CRT (Multicenter Automatic Defibrillator Implantation Trial-Cardiac Resynchronization Therapy): Cardiac resynchronization therapy towards early management of heart failure. Eur. Heart J..

[29] Sauer W.H., Bristow M.R. (2008). The Comparison of Medical Therapy, Pacing, and Defibrillation in Heart Failure (COMPANION) trial in perspective. J. Interv. Card. Electrophysiol..

[30] Cleland J.G., Freemantle N., Erdmann E., Gras D., Kappenberger L., Tavazzi L., Daubert J. (2012). Long-term mortality with cardiac resynchronization therapy in the Cardiac Resynchronization-Heart Failure (CARE-HF) trial. Eur. J. Heart Fail..

[31] Januzzi J.L., Butler J., Fombu E., Maisel A., McCague K., Piña I.L., Prescott M.F., Riebman J.B., Solomon S. (2018). Rationale and methods of the Prospective Study of Biomarkers, Symptom Improvement, and Ventricular Remodeling During Sacubitril/Valsartan Therapy for Heart Failure (PROVE-HF). Am. Heart J..

[32] Strisciuglio T., Ammirati G., Pergola V., Imparato L., Carella C., Koci E., Chiappetti R., Abbate F.G., La Fazia V.M., Viggiano A. (2019). Contrast-induced nephropathy after cardiac resynchronization therapy implant impairs the recovery of ejection fraction in responders. ESC Heart Fail..

[33] Mann D.L., Givertz M.M., Vader J.M., Starling R.C., Shah P., McNulty S.E., Anstrom K.J., Margulies K.B., Kiernan M.S., Mahr C. (2022). Effect of Treatment with Sacubitril/Valsartan in Patients with Advanced Heart Failure and Reduced Ejection Fraction. JAMA Cardiol..

[34] Mentz R.J., Ward J.H., Hernandez A.F., Lepage S., Morrow D.A., Sarwat S., Sharma K., Starling R.C., Velazquez E.J., Williamson K.M. (2023). Angiotensin-Neprilysin Inhibition in Patients with Mildly Reduced or Preserved Ejection Fraction and Worsening Heart Failure. J. Am. Coll. Cardiol..

